# The effects of different hormone combinations on the growth of *Panax notoginseng* anther callus based on metabolome analysis

**DOI:** 10.3389/fpls.2024.1503931

**Published:** 2024-12-09

**Authors:** Saiying Yu, Leilin Li, Tiantai Liu, Jianbin Li, Qian Yang, Xiuming Cui

**Affiliations:** ^1^ Faculty of Life Science and Technology, Kunming University of Science and Technology, Kunming, China; ^2^ Key Laboratory of Panax notoginseng Resources Sustainable Development and Utilization of State Administration of Traditional Chinese Medicine, Kunming, China; ^3^ Yunnan Provincial Key Laboratory of Panax notoginseng, Kunming, China; ^4^ Kunming Key Laboratory of Sustainable Development and Utilization of Famous-Region Drug, Kunming, China; ^5^ Sanqi Research Institute of Yunnan Province, Kunming, China

**Keywords:** auxin response factor, cytokinin response factor, *P. notoginseng* callus, saponin synthesis, secondary metabolite, TCA cycle

## Abstract

*Panax notoginseng* saponins (PNS), the primary active components of *Panax notoginseng* (Burk.) F.H.Chen, a traditional and precious Chinese medicinal herb, are mainly derived from the roots of the plant. However, due to the long cultivation period and specific environmental requirements, the PNS supply is often limited. And, callus cultures of *P. notoginseng*, which grow rapidly, have short production cycles, and can be cultured under controlled conditions, provide a more efficient source for the quick acquisition of saponins. In this study, anthers of *P. notoginseng* were used as explants, and twelve hormone combinations were tested to induce callus formation. Eight kinds of hormone combinations successfully induced *P. notoginseng* anther callus. Among these, callus induced by combinations 5 and 7 had the highest saponin content, while those induced by combinations 1 and 3 exhibited the highest relative growth rates. Metabolomic analysis of these four callus types revealed that there were a total of 99 differential metabolites between combinations 5 and 7, 30 between combinations 1 and 3, 123 between combinations 3 and 7, and 116 between combinations 1 and 5. Further analysis showed that the tricarboxylic acid (TCA) cycle metabolites in callus induced by combinations 1 and 3 were significantly upregulated, with corresponding genes showing high expression levels, increased ATP accumulation, and low responses of the auxin response factor *PnARF-3* and cytokinin response factor *PnCRF-3*. The abundance of metabolites in the PNS biosynthesis pathway in callus induced by combinations 5 and 7 increased significantly, with related genes showing high expression levels, increased IPP accumulation, and high responses of *PnARF-3* and *PnCRF-3*. Overexpression of *PnARF-3* and *PnCRF-3* in callus induced by combination 3 promoted the production of IPP and saponins while reducing ATP production. In conclusion, different hormone combinations affect the distribution of Acetyl-CoA through *PnARF-3* and *PnCRF-3*, resulting in the relative growth rate and saponin of *P. notoginseng* anther callus differences.

## 1 Introduction

**Figure 1:**
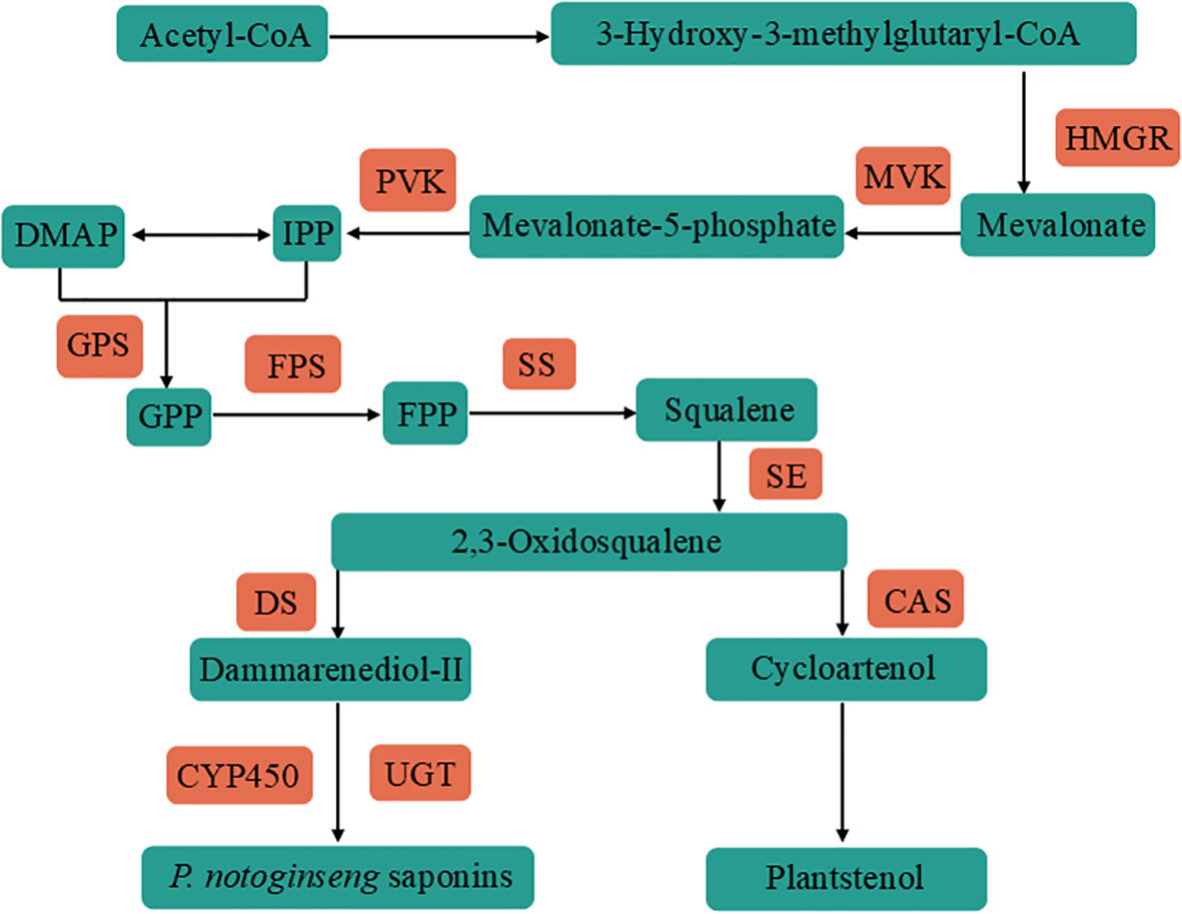
Flow chart of PNS synthesis, with Acetyl-CoA as the starting material. The precursor isopentenyl pyrophosphate (IPP) was synthesized by 3-Hydroxy-3-methylglutaryl-CoA reductase (HMGR), mevalonate kinase (MVK) and phosphomevalonate kinase (PVK). IPP was via geranyl pyrophosphate synthase (GPS), farnesyl diphosphate synthase (FPS), squalene synthase (SS) and squalene epoxidase (SE) synthetic skeleton 2,3-oxidosqualene. 2,3-oxidosqualene is synthesized PNS by Dammarenediol-II synthase (DS), CYP450 and UGT. The blue boxes denote the substances involved in the synthesis process, while the red boxes indicate the key genes.

*Panax notoginseng* (Burk.) F.H.Chen is a perennial herbaceous plant belonging to the genus Panax in the family Araliaceae, with a long history of cultivation. It is mainly cultivated in Wenshan City, Yunnan Province (Park et al., 2012), and is a unique and precious Chinese herbal medicine. *Panax notoginseng* saponins (PNS) are widely recognized as the primary medicinal components of *P. notoginseng*. Approximately one hundred saponins have been identified in *P. notoginseng* (Wang et al., 2016), predominantly belonging to the dammarane-type tetracyclic triterpenes. Among these, the most prevalent are five monomeric saponins namely notoginsenoside R1, ginsenoside Rg1, ginsenoside Rb1, ginsenoside Re and ginsenoside Rd. Additionally, *P. notoginseng* contains several rare saponins such as ginsenoside Rc and ginsenoside Rg2 (Hao et al., 2022). The upstream precursor synthesis pathway of PNS primarily involve mevalonic acid (MVA) and methylerythritol (MEP) pathway (Cun et al., 2024). The MVA pathway uses Acetyl-CoA as a substrate to synthesize mevalonate under the action of 3-hydroxy-3-methylglutaryl-CoA reductase (HMGR). Then mevalonate is converted into isopentenyl pyrophosphate (IPP) through the actions of mevalonate kinase (MVK) and phosphomevalonate kinase (PVK). IPP further synthetizes dimethylallyl pyrophosphate (DMAPP), while the MEP pathway also generates IPP and DMAPP (Singh et al., 2023; Liu et al., 2020). In the intermediate stage of PNS biosynthesis, IPP and DMAPP are converted into 2,3-oxidosqualene by the actions of geranyl pyrophosphate synthase (GPS), farnesyl diphosphate synthase (FPS), and squalene epoxidase (SE). Afterwards, Dammarenediol-II synthase (DS) and cycloartenol synthase (CAS) cyclize 2,3-oxidosqualene into dammarenediol-II and cycloartenol. Dammarenediol-II is further hydroxylated, oxidized and glycosylated by cytochrome P450 monooxygenase (CYP450) and UDP-glucosyltransferase (UGT) to complete the synthesis of PNS (**Figure 1**) (Xu et al., 2018; Li et al., 2022).

At present, biosynthesis technology is developing rapidly and a yeast factory for the synthesis of PNS has been initially constructed, but produces a limited variety of monomeric saponins with extremely low yields. The main raw material of PNS is still the *P. notoginseng* plant at this stage. The artificial cultivation of *P. notoginseng* has a long history, and people have accumulated extensive experience through long-term production practice. However, its cultivation is limited by several factors, including the lack of effective breeding techniques, a long growth cycle, poor ecological adaptability, and a narrow geographical distribution. Additionally, continuous cropping obstacles (Yang et al., 2015), soil microorganisms (Zhao et al., 2020), and various diseases and pests further complicate the cultivation process. The limited planting area of *P. notoginseng* cannot meet the market demand for PNS, hindering its application. Plant tissue culture technology provides a solution to this problem. By inducing *P. notoginseng* callus, a large quantity of raw materials for extracting PNS can be produced. This not only helps to quickly solve the problem of shortage of PNS, but also find a new way for the production of secondary metabolites.

In recent years, the callus induction system of many kinds of medicinal plants has been well developed, such as *P. ginseng*, *P. quinquefolius*, *Alfalfa*, *Macleaya cordata*, *Angelica dahurica*, *Forsythia suspensa*, *Lycium barbarum* and *Weigela florida ‘Red Prince’* (**Supplementary Table S1**) (Lei, 2013; Yin, 2015; Cui, 2017; Song, 2014; Li, 2013; Ren, 2009; Luo et al., 2016; Wang, 2012). A large number of studies have shown that the primary factors influencing callus quality are the ratio and concentration of plant hormones. Lei (2013) investigated the effects of different hormone combinations on the callus induction rate and total saponin content of P. ginseng anthers. The study found that the combination of 2,4-D (1.5 mg/L) and KT (0.5 mg/L) achieved the highest callus induction rate, while the total content of six monomeric saponins (ginsenoside Rg1, Re, Rb1, Rc, Rb2, and Rd) was only 0.112%. In comparison, the combination of 2,4-D (1.0 mg/L) and 6-BA (0.5 mg/L) resulted in a higher total saponin content of 0.258%, despite not achieving the highest callus induction rate. The research found that the total amount of six monomeric saponins (ginsenoside Rg1, ginsenoside Re, ginsenoside Rb1, ginsenoside Rc, ginsenoside Rb2, ginsenoside Rd) in anther callus of *P. quinquefolius* reached 3.26%. However the callus growth rate was relatively lower, only 153%, under the hormone combination of 2,4-D (2.0 mg/L) and 6-BA (0.5 mg/L). Under the combination of 2,4-D (2.0 mg/L) and 6-BA (2.0 mg/L), the content of six monomeric saponins was only 0.15%, but the callus had the highest growth rate (212%) (Zhang, 2013). The research showed that the hormone ratio had the greatest effect on the content of saponins in *P. notoginseng* callus. Under the combination of 2,4-D (0.5 mg/L) and 6-BA (1.0 mg/L), the total amount of ginsenoside Rg1, ginsenoside Re, ginsenoside Rb1 and ginsenoside Rd in the callus was the highest. However, how these hormones affected the callus induction rate and growth rate, and the reason for the content of secondary metabolites was unclear (Qi et al., 2017).

In this study, *P. notoginseng* anther was used as the explant to induce callus formation. The morphology and structure between embryogenic calli and non-embryogenic callus were observed. The effects of different hormone combinations on the induction rate, relative growth rate and saponins content of *P. notoginseng* anther callus were studied. Using metabolomics and RT-qPCR analysis, we screened the hormone combinations that led to the highest saponin accumulation in *P. notoginseng* anther callus, as well as the key genes *P. notoginseng* auxin response factors 3 (*PnARF-3*) and cytokinin response factors 3 (*PnCRF-3*) involved in saponin synthesis. Finally, auxin response factor *PnARF-3* and cytokinin response factor *PnCRF-3* were overexpressed in *P. notoginseng* anther callus by transient expression system. Different hormone combinations were found to influence Acetyl-CoA distribution via *PnARF-3* and *PnCRF-3*. Overexpression of *PnARF-3* and *PnCRF-3* promotes saponins accumulation while reducing ATP production. This provides an effective approach for the production of PNS, with significant economic and commercial value.

## 2 Materials and methods

### 2.1 Plant materials

**Figure 2:**
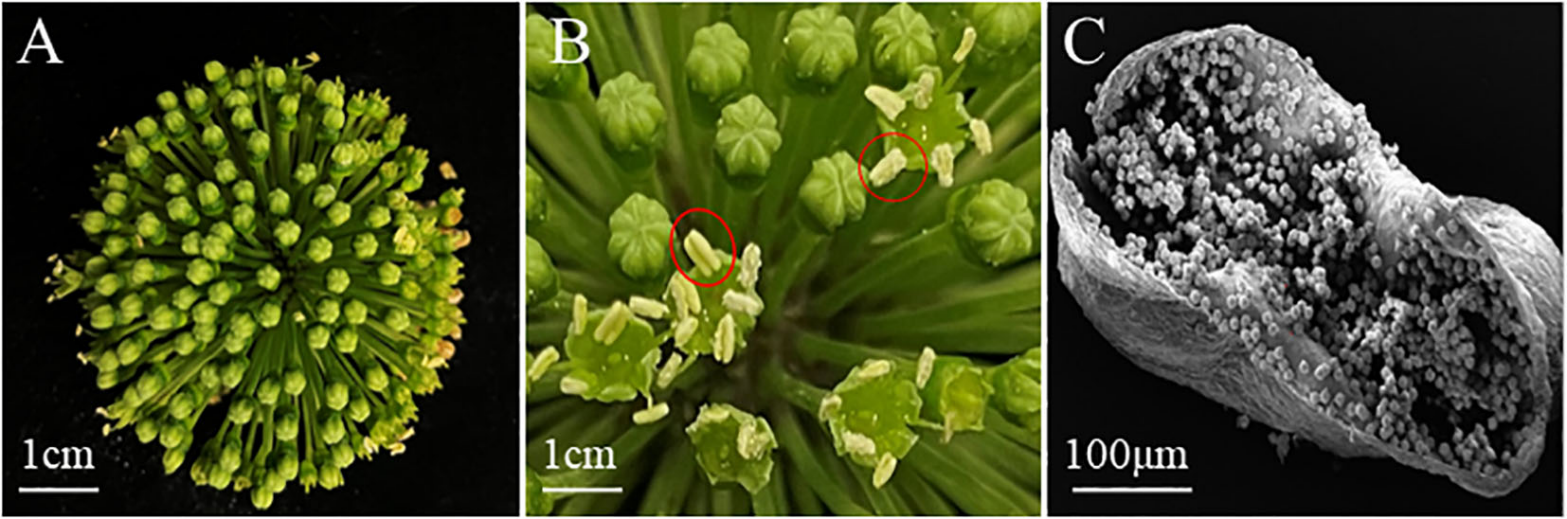
The appearance and structure of *P. notoginseng* flower, bud and anther. **(A)** *P. notoginseng* Flower; **(B)** Bud, within the red circle is the anther; **(C)** The scanning electron microscope result of anther was columnar.

In this study, the anther of *P. notoginseng* was used as explants. Unpollinated flower buds were collected and anthers were extracted from them (**Figure 2B**). The scanning electron microscope result of anther was columnar (**Figure 2C**). The *P. notoginseng* flower (**Figure 2A**) was collected from the planting base of *P. notoginseng* in Wenshan City, Yunnan Province (E 104.09◦, N 23.4◦).

### 2.2 Induction of callus in anther of *P. notoginseng*

The buds of *P. notoginseng* were pretreated at low temperature for 7 days. Under sterile conditions, the buds of *P. notoginseng* soaked in ethanol (75%, v/v; 30s), sodium hypochlorite (NaClO) (10%, m/w; 5 min), and sterile water (5 times). The surface of the buds was dried with sterile filter paper, and the anthers were removed with sterile tweezers and inoculated into different hormone combinations (**Supplementary Table S1**) (Bansal et al., 2022; Jin et al., 2022). The cultures were kept in the dark at 25 ± 1°C and 50% humidity, and the callus induction rate was measured after 50 days. The induction rate of anther callus (%) = the number of anthers with callus/the total number of anthers inoculated.

### 2.3 Determination of relative growth rate of anther callus of *P. notoginseng*

Callus measuring 3–4 cm in length was cut into square of approximately 1cm, then subcultured, and the relative growth rate of callus was measured.

Callus relative growth rate (CRGR) was evaluated by the following formula: CRGR = (lnW2 − lnW1)/number of days (Huang et al., 2023).

Where W1 and W2 represent the average fresh weight at day 0 and culture day, respectively.

### 2.4 Determination of saponins content in the anther callus of *P. notoginseng*

The callus under different hormone combinations No.1-No.8 was dehydrated using a vacuum freeze dryer, then ground into powder. 0.1 g of the powder was weighed and mixed with 0.5 mL of methanol, followed by soaking at room temperature for 24 hours. The mixture was then ultrasonicated at 200 W and 50 kHZ for 40 min, and centrifuged at 8000 rpm for 5 min. The supernatant was collected, filtered with a 0.45 μM organic filter, and used to prepare the test sample. Additionally, 3.1 mg of each standard (notoginsenoside R1, ginsenoside Rg2, Rb1, Rg1, Rd, Rc and Re) was weighed, dissolved in 1 mL of methanol, filtered with a 0.22 μM organic filter, and prepared as the test sample.

The concentrations of notoginsenoside R1, ginsenoside Rg2, ginsenoside Rb1, ginsenoside Rg1, ginsenoside Rd, ginsenoside Rc and ginsenoside Re was analyzed by an HPLC system (Shimadzu, LC-40D). The mobile phase consisted of ultra-water (A) and acetonitrile (B) and was run in an isocratic mode at a flow rate of 1.0 mL/min. The following gradient was performed: 0-30 min, 20% B; 30-62 min, 47% B; 62-80 min, 80% B; 80-85 min, 100% B; 85-95 min, 100% B; 95-96 min, 20% B; 96-110 min, 20% B. The column temperature was maintained at 35°C. Aliquots of 20 µL were injected. Monitoring and quantitation of notoginsenoside R1, ginsenoside Rg2, ginsenoside Rb1, ginsenoside Rg1, ginsenoside Rd, ginsenoside Rc and ginsenoside Re were performed at 203 nm.

### 2.5 Paraffin sections of anther callus of *P. notoginseng*

The callus in different hormone combinations were first fixed in FAA fixative solution, then washed and dehydrated with different concentrations of ethanol. The samples were then cleared with a mixture of ethanol and dimethylbenzene, infiltrated with a mixture of dimethylbenzene and paraffin, and embedded in paraffin cubes. The embedded samples were sliced to a thickness of 7 μM. The slices were first stained with saffron dye, then decolorized, and subsequently stained with solid green dye. After dehydration, the samples were cleared with xylene and mounted with neutral gum. The histomorphological structure of *P. notoginseng* anther callus was observed using a microscope (Ellison et al., 2016; Huang et al., 2023).

### 2.6 Anther callus metabolome of *P. notoginseng*

The callus samples of two hormone combinations with the highest relative growth rate (No.1 and No.3) and two hormone combinations with the highest content of total saponins, including 7 monomers (No.5 and No.7) were selected for metabolic analysis. **Supplementary Method S1** provides detailed information on the extraction, detection, identification, quantification and statistics of differential metabolites.

### 2.7 Determination of ATP and IPP in the anther callus of *P. notoginseng*

The contents of adenosine triphosphate (ATP) and isopentene pyrophosphate (IPP) in callus with different hormone combinations (No.1, No.3, No.5, No.7) were detected by a kit (Solarbio, China) according to the manufacturer’s recommended procedure.

### 2.8 RT-qPCR detection of *P. notoginseng* anther callus

The total RNA was extracted from the callus of *P. notoginseng* anther using Trizol reagent (Takara) with different hormone combinations (No.1, No.3, No.5, No.7), and 1μg RNA was reverse transcribed into cDNA by Prime Script RT kit (with 8 × gDNA Eraser Premix, 5 × RT Premix, RNase Free H_2_O, Takara). Prime 5.0 software was used to design RT-qPCR gene specific primers. *PnACTIN2* gene of *P. notoginseng* was used as the internal reference gene, the relative gene expression level was calculated by 2^-ΔΔCT^ method (Livak and Schmittgen, 2001).

### 2.9 Agrobacterium-mediated overexpression of *PnARF-3* and *PnCRF-3* in anther callus of *P. notoginseng*

The overexpression vectors were transformed into the callus of hormone combination No.3 using a transient expression system constructed by Xian et al. (2023). RNA was extracted from the callus and converted into cDNA, which was used to amplify the *PnARF-3* and *PnCRF-3* genes for constructing the overexpression vectors pBWA(V)HS*-PnARF-3* and pBWA(V)HS*-PnCRF-3*. These vectors were transformed into *Escherichia coli* competent cells and the recombinant plasmids were then extracted. These plasmids were subsequently transformed into competent GV3101 Agrobacterium cells.

### 2.10 Statistical analysis

All the experiments were repeated three times, and the statistical significance was analyzed by Prism software (GraphPad Software).

## 3 Results

### 3.1 Callus induction in *P. notoginseng* anthers with different hormone combinations

Among the 12 hormone combinations tested, combinations No.1 to No.8 successfully induced callus formation from *P. notoginseng* anther, whereas combinations No.9 to No.12 failed to induce callus (**Supplementary Table S3**). As a result, further studies were conducted with a focus on the callus induced by hormone combinations No.1 - No.8.

### 3.2 Morphological characteristics of callus with different hormone combinations

**Figure 3:**
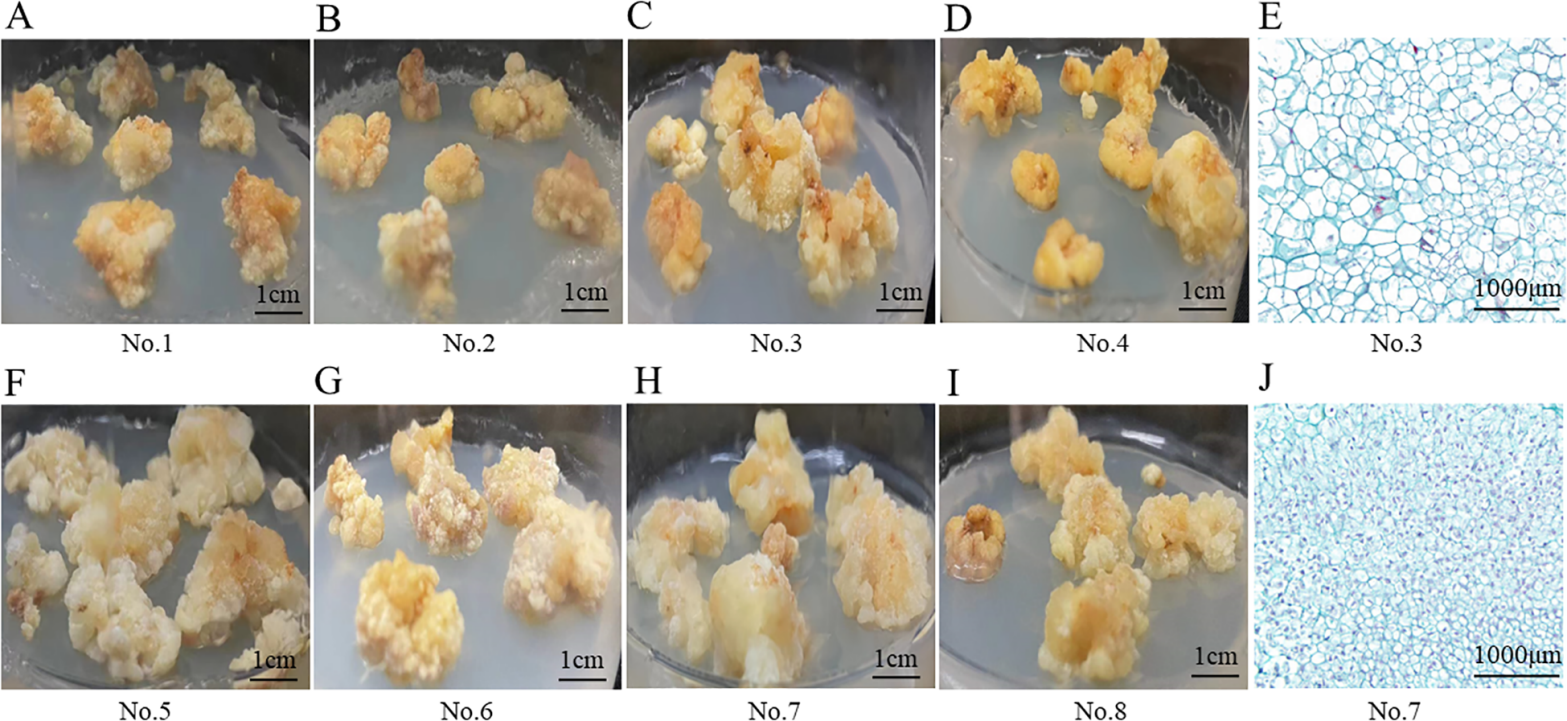
Morphological structure of *P. notoginseng* anther callus with different hormone combinations. **(A–D)** Appearance structure of *P. notoginseng* anther callus with hormone combinations No.1-No.4; **(E)** Observation of cytological structure of non-embryogenic callus by microscope (No.3); **(F–I)** Appearance structure of *P. notoginseng* anther callus with hormone combinations No.5-No.8; **(J)** Observation of cytological structure of embryogenic callus by microscope (No.7).

During the callus induction process, two distinct types of callus morphology were observed: embryogenic callus and non- embryogenic callus. Among the eight hormone combinations tested, combinations No.1 to No.4 induced non-embryogenic callus, whereas combinations No.5 to No.8 resulted in embryogenic callus. The non-embryogenic callus displayed irregular shapes, a loose texture, and a rough surface with minor protrusions, and was either bright yellow or pale yellow in appearance (**Figures 3A–D**). Microscopic examination revealed that the nuclei were extremely small, the vacuoles occupied more than 85% of the cell area, and the cell had irregular shapes and loosely arranged (**Figure 3E**). In contrast, the embryogenic callus had a compact texture and appeared yellow-white (**Figures 3F–I**). Under the microscope, the cells presented that they were densely packed, uniform in size, with a large stained area and dense cytoplasm (**Figure 3J**).

Additionally, paraffin sections revealed that calluses treated with the same hormone combinations exhibited identical shapes. For instance, calluses for hormone combinations No.1 and No.2 consistently displayed heart-shaped embryos (**Supplementary Figures S1A, B**). The calluses form combinations No.3 and No.4, No.5 and No.6, as well as No.7 and No.8, also exhibited similar shapes (**Supplementary Figures S1C–H**). However, significant differences in callus morphology were observed among the various hormone combinations. For example, the calluses form No.3 and No.5 displayed markedly different shapes (**Supplementary Figures S1C, E**).

### 3.3 Different hormone combinations had significant effects on callus induction rate and relative growth rate of *P. notoginseng* anther

**Figure 4:**
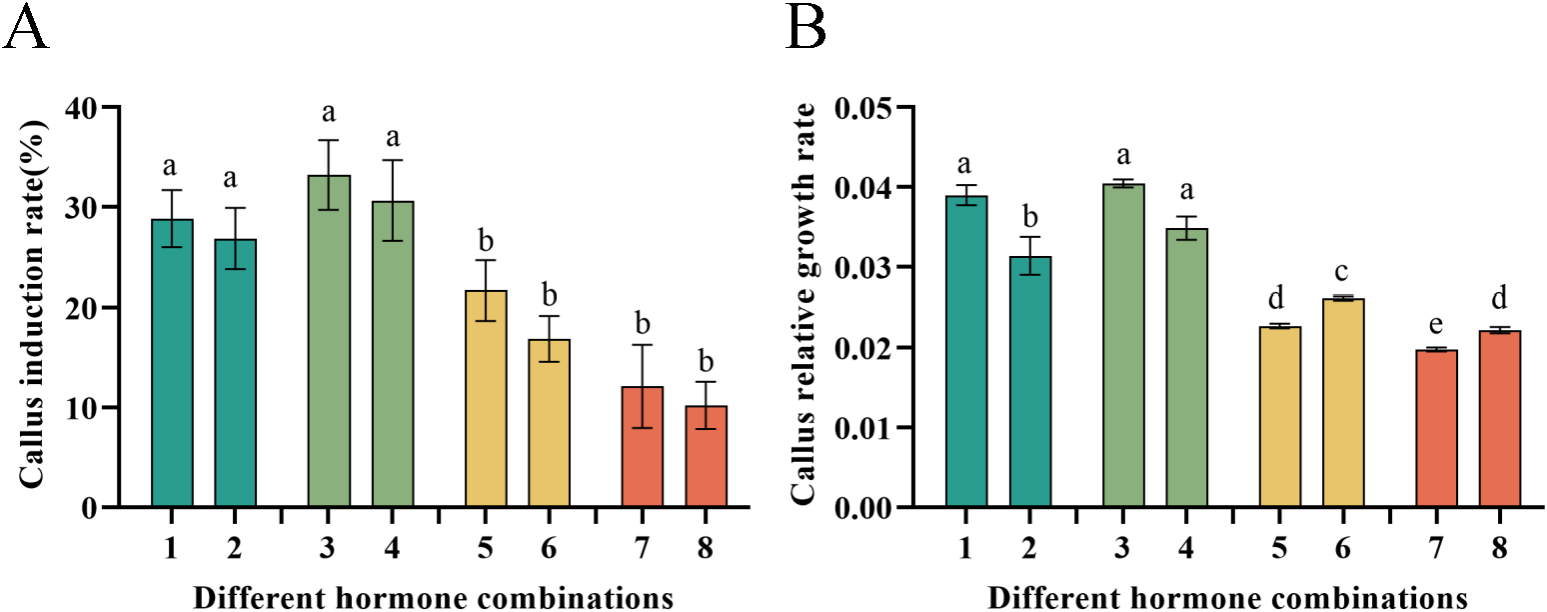
Illustrates the callus induction rate and relative growth rate of *P. notoginseng* anther with different hormone combinations. **(A)** Callus induction rate of *P. notoginseng* anther in hormone combination No.1-No.8; **(B)** Callus relative growth rate of *P. notoginseng* anther in hormone combination No.1-No.8. Data are expressed as average values ± SE (n=3), different letters indicate significant differences at p < 0.05 (Student’s test).

Significant differences were observed in the induction rate of *P. notoginseng* anther callus among different types and concentrations. Combinations No.3 and No.4 (2,4-D + 6-BA + NAA + KT) achieved the highest induction rates, at 33.22% and 30.66%, respectively. The induction rates for hormone combinations No.1 and No.2 (2,4-D + KT + IAA) were 28.85% and 26.84%, respectively. Combinations No.5 and No.6 (2,4-D + 6-BA) yielded induction rates of 21.69% and 16.89%. In contrast combinations No.7 and No.8 (2,4-D + KT + NAA) produced the lowest induction rates at 12.11% and 10.22%, respectively (**Figure 4A**). Additionally, different hormone combinations had a significant impact on the relative growth rates of the *P. notoginseng* anther callus. The relative growth rates were ordered from highest to lowest as follows: No.3, No.4 > No.1, No.2 > No.5, No.6 > No.7, No.8 (**Figure 4B**). Among these, the relative growth rate of hormone combination No.3 achieved the highest of 0.0405. Combinations No.1, No.4 and No.2 had relative growth rates of 0.0390, 0.0348, and 0.0314, respectively. Combinations No.6, No.8 and No.5 had relatively similar growth rates of 0.0262, 0.0229, and 0.0227, respectively. Combination No.7 exhibited the lowest relative growth rate of 0.0198, which was 104.55% lower than that of combination No.3.

### 3.4 Effect of different hormone combinations on saponin accumulation in *P. notoginseng* anther callus

**Figure 5:**
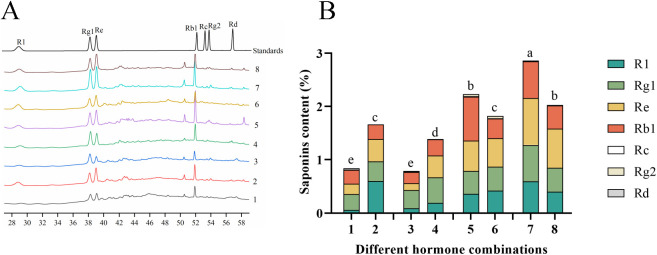
Displays the saponin content in Callus of *P. notoginseng* anther with different hormone combinations. **(A)** HPLC chromatogram of *P. notoginseng* anther callus in hormone combination No.1-No.8; **(B)** The content of total saponins of seven monomers in *P. notoginseng* anther callus in hormone combination No.1-No.8 was determined by HPLC. Different letters indicate significant differences at p < 0.05 (Student’s test).

Since saponins are the primary active constituents of *P. notoginseng*, we assessed the levels of seven saponin monomers in the anther callus under different hormone combinations (**Figure 5A**). The results revealed that the contents of notoginsenoside R1, ginsenoside Rg1, ginsenoside Re and ginsenoside Rb1 were the most in the anther callus, whereas the contents of ginsenoside Rc, ginsenoside Rg2 and ginsenoside Rd were relatively low (**Supplementary Table S3**). This distribution is consistent with the main saponins found in the intact *P. notoginseng* plant. The highest total saponin content was observed in combination No.7 (**Figure 5B**), at 2.8624%, which was 3.6 times higher than the lowest content found in combination No.3. Combinations No.5, No.8 and No.6 had total saponin contents of 2.2369%, 2.0245% and 1.8088%, respectively. Combinations No.2 and No.4 had similar total saponin contents of 1.6650% and 1.5648%, respectively. The lowest total saponin contents were found in combinations No.1 and No.3, at 0.8491% and 0.7953%, respectively. These results suggest that the primary saponin types in the *P. notoginseng* anther callus are similar to those in the intact plant, and that the saponin content is influenced by the hormone combinations used.

### 3.5 Metabolome analysis of the effects of different hormone combinations on the callus metabolites of *P. notoginseng* anthers

**Figure 6:**
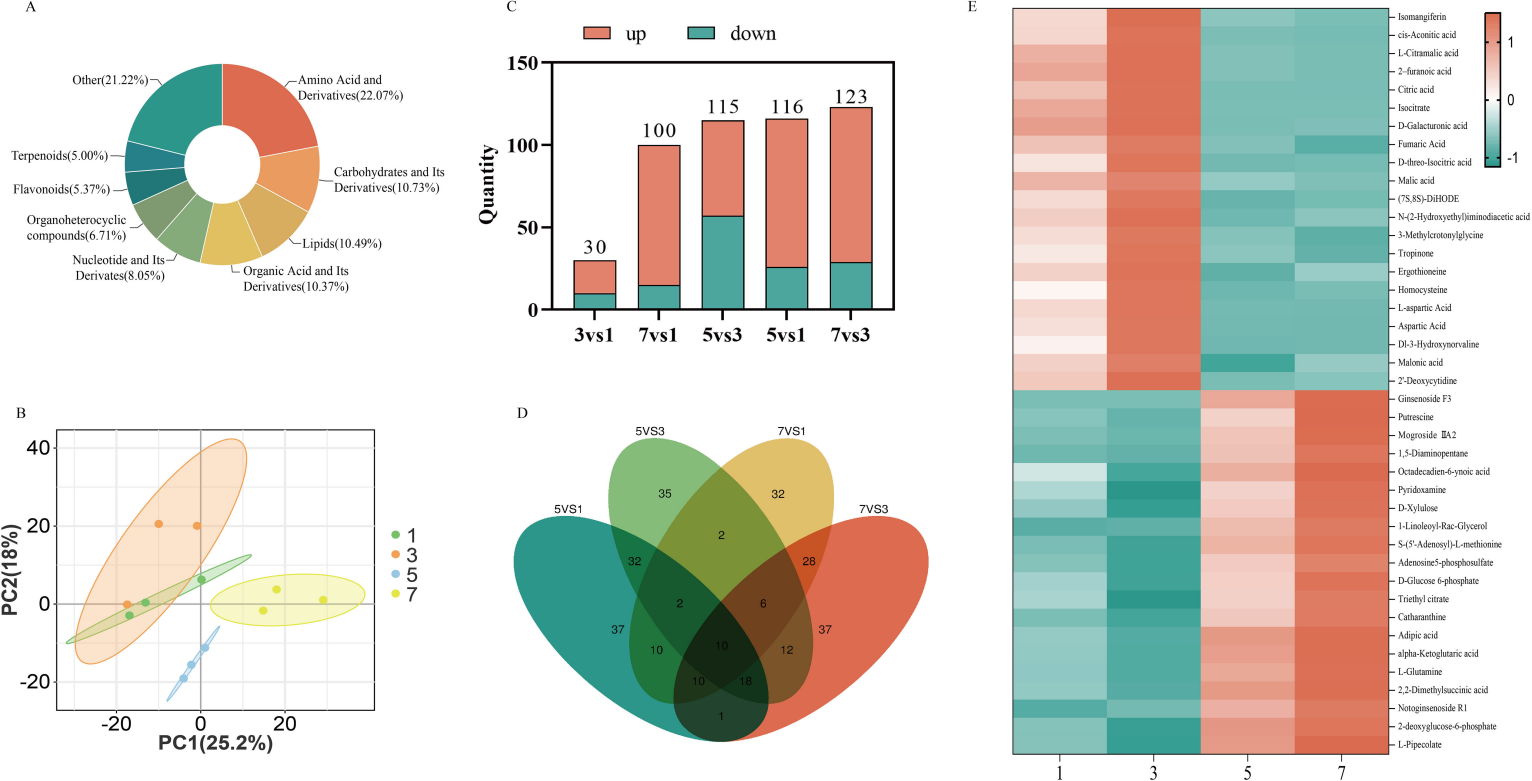
Analysis the metabolite of *P. notoginseng* anther callus with different hormone combinations; **(A)** The proportion of different metabolites in the anther callus of *P. notoginseng*; **(B)** PCA principal component analysis diagram of different hormone combinations; **(C)** The number of different metabolites in different comparison groups; **(D)** Venn diagram; **(E)** The cluster heat map of the 20 metabolites of the most up-regulated and down-regulated in four hormone combinations.

To investigate the impact of hormone combinations on the metabolic levels of *P. notoginseng* anther callus, we selected four hormone combinations for metabolomic analysis: the two with the highest saponin content (No.5 and No.7) and the two with the fastest relative growth rates (No.1 and No.3). A total of 820 metabolites were detected, including 181 amino acids and their derivatives, 88 carbohydrates and their derivatives, 85 organic acids and their derivatives, and 41 terpenes (**Figure 6A**). PCA analysis revealed clear separations between the different hormone combinations, indicating significant metabolic changes due to the hormone treatments (**Figure 6B**). Differential metabolite analysis showed that only 30 differential metabolites were identified in the comparison between combinations No.3 and No.1, whereas 116, 115, 100, and 123 differential metabolites were found in the comparisons of No.5 vs No.1, No.5 vs No.3, No.7 vs No.1, and No.7 vs No.3, respectively (**Figure 6C**). This indicates substantial differences in metabolites among the anther callus samples from different hormone combinations, with the least variation observed between No.1 and No.3. The Venn diagram showed that only 10 metabolites were common to all four hormone combinations (**Figure 6D**), further emphasizing the differences in metabolites under varying hormone treatments. Despite the highly similar metabolite profiles between No.1 and No.3, cluster heatmap analysis of the top 20 upregulated and downregulated metabolites in the comparisons of No.5 vs No.3 and No.7 vs No.3 revealed that substances such as cis-aconitic acid, citric acid, isocitrate, fumaric acid, and malic acid were highly expressed in combination No.3, also significantly upregulated in combination No.1, but downregulated in combinations No.5 and No.7. Conversely, notoginsenoside R1 and ginsenoside F3 were highly expressed in combination No.7, significantly upregulated in combination No.5, but notably downregulated in combinations No.1 and No.3 (**Figure 6E**).

**Figure 7:**
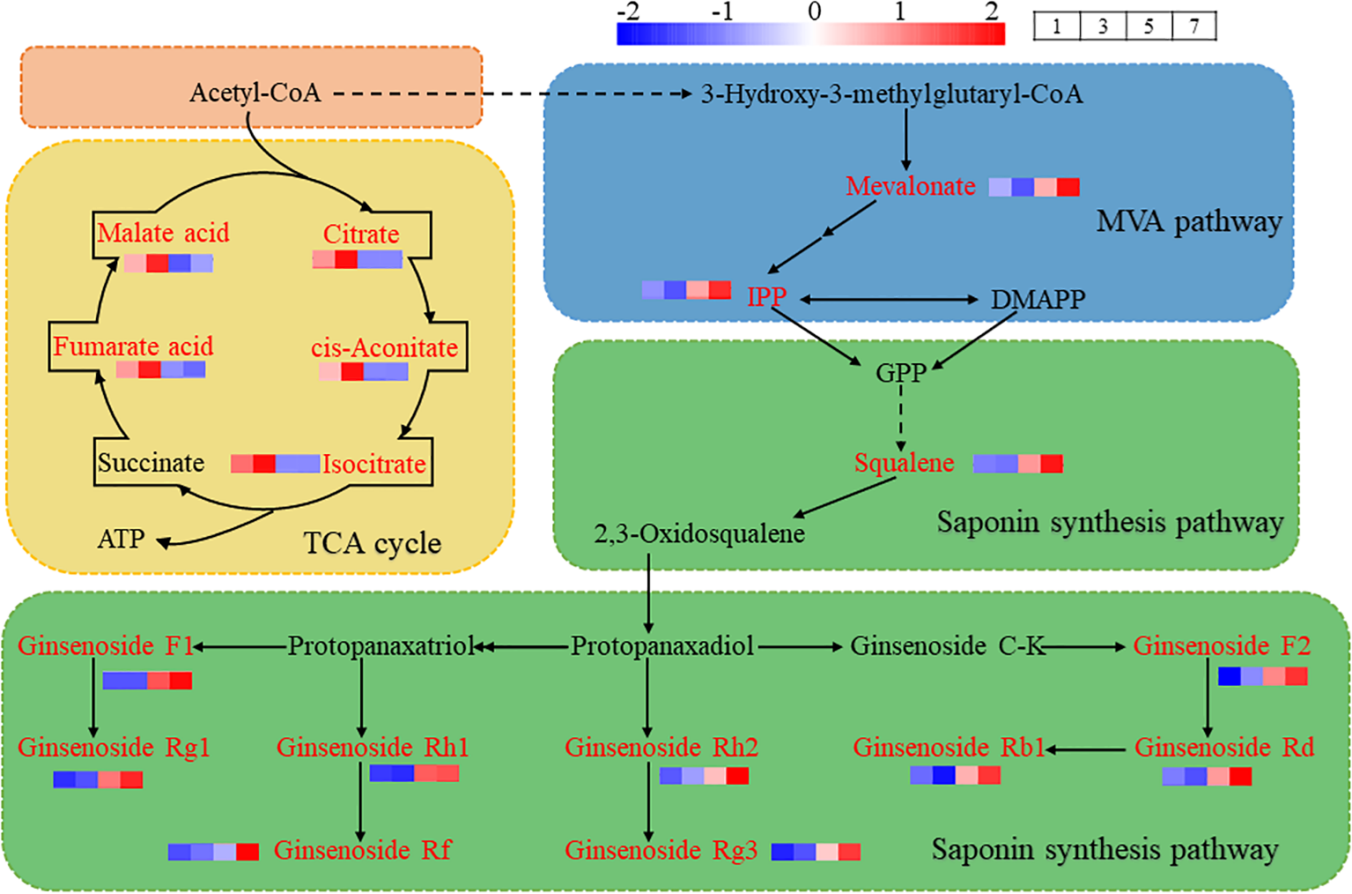
Illustrates the TCA cycle, MVA Pathway and downstream of saponins synthesis metabolic pathway. Acetyl-CoA serves as a precursor, part of it enters the TCA cycle to synthesize citrate acid, cis-aconitate acid, isocitrate, fumarate acid, malate acid and other substances to produce ATP. Some of these substances are directed into the MVA pathway to generate mevalonate, IPP and 2,3-Oxidosqualene. 2,3-Oxidosqualene entered the saponin synthesis pathway, and further synthesized ginsenoside Rg1, ginsenoside Rf, ginsenoside Rg3, ginsenoside Rd and ginsenoside Rb1. The red box highlights precursor substances, the yellow box denotes TCA cycle, the blue box represents MVA pathway, and the green box indicates downstream pathway of saponin synthesis. The red font identifies the detected metabolites in the callus of *P. notoginseng* anther, while the black font denotes the undetected metabolites in the callus of *P. notoginseng* anther.

Further analysis revealed that cis-aconitate acid, citrate acid, isocitrate, fumarate acid and malate acid, which were down-regulated in the comparisons of No.5 vs No.3 and No.7 vs No.3, are the key substances in the tricarboxylic acid (TCA) cycle. The up-regulated differential metabolites, notoginsenoside R1 and ginsenoside F3 are products of MVA pathway and downstream pathway of saponins synthesis. The TCA cycle and MVA pathway share a common precursor, Acetyl-CoA, suggesting that different hormone combinations affect the distribution of Acetyl-CoA. In hormone combination No.1 and No.3, more Acetyl-CoA entered the TCA cycle, while hormone combinations No.5 and No.7 directed more Acetyl-CoA into the MVA pathway (**Figure 7**).

### 3.6 Effects of different hormone combinations on ATP and IPP contents of *P. notoginseng* anther callus

**Figure 8:**
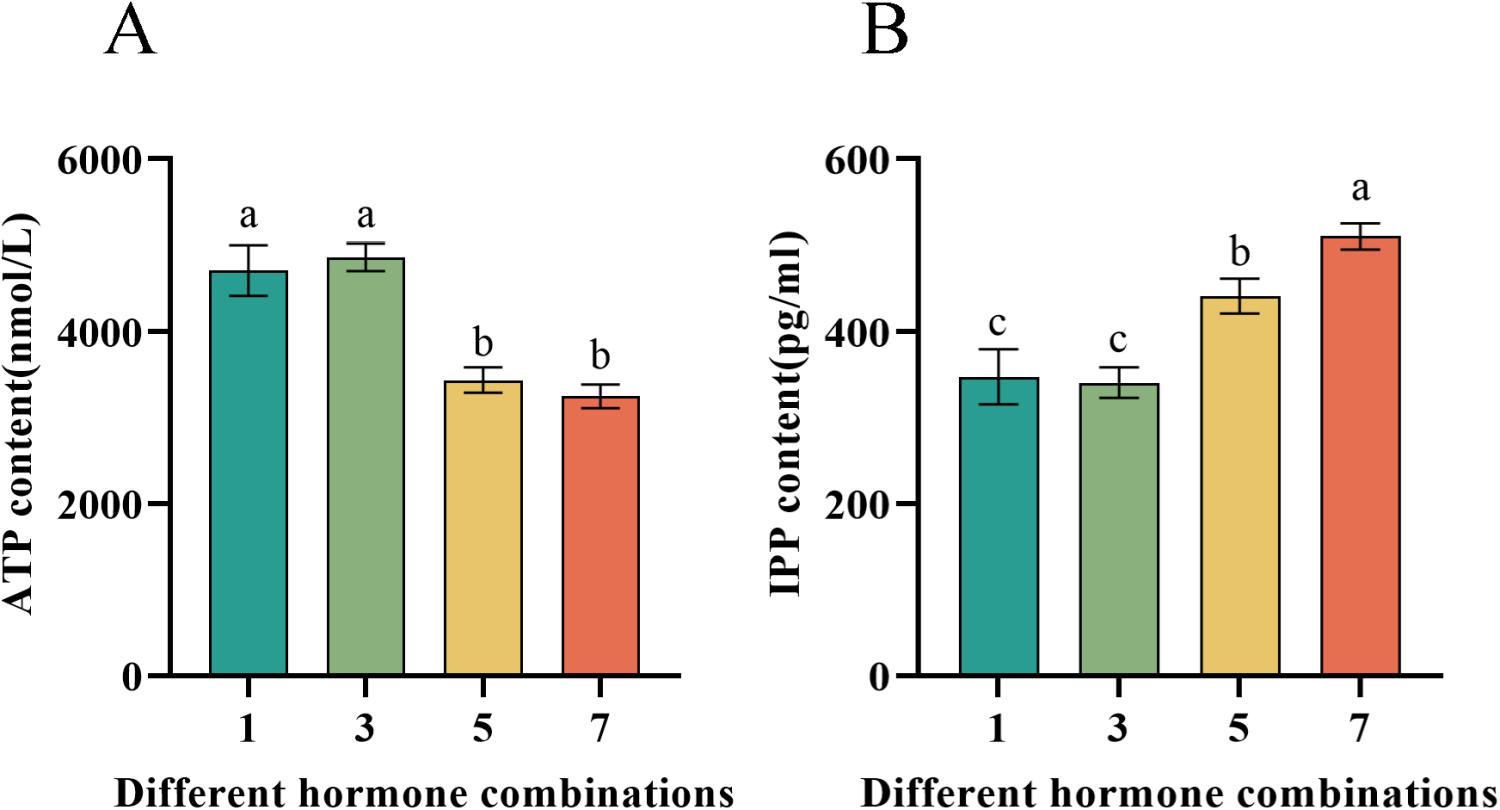
Presents the contents of ATP and IPP in callus of *P. notoginseng* anther with different hormone combinations. **(A)** Determination of ATP content of *P. notoginseng* anther callus in different hormone combinations by kit; **(B)** Determination of IPP content of *P. notoginseng* anther callus in different hormone combinations by kit. Data are expressed as average values ± SE (n=3), different letters indicate significant differences at p < 0.05 (Student’s test).

The metabolomic analysis revealed significant differences among the four hormone combinations concerning the TCA cycle and the upstream MVA pathway of saponin synthesis. Consequently, we measured the levels of ATP, a product of the TCA cycle, and IPP, a product of the MVA pathway. The results indicated that hormone combinations No.1 and No.3 significantly increased ATP levels in the *P. notoginseng* anther callus (**Figure 8A**), thus providing more energy and promoting growth. On the other hand, hormone combinations No.5 and No.7 significantly increased IPP levels in the *P. notoginseng* anther callus, thereby enhancing saponin synthesis, with combination No.7 having a greater effect than combination No.5 (**Figure 8B**). These findings are consistent with the metabolite analysis results.

### 3.7 The expression levels of genes related to the TCA cycle and saponin biosynthesis in the callus varied significantly under four hormone combinations

**Figure 9:**
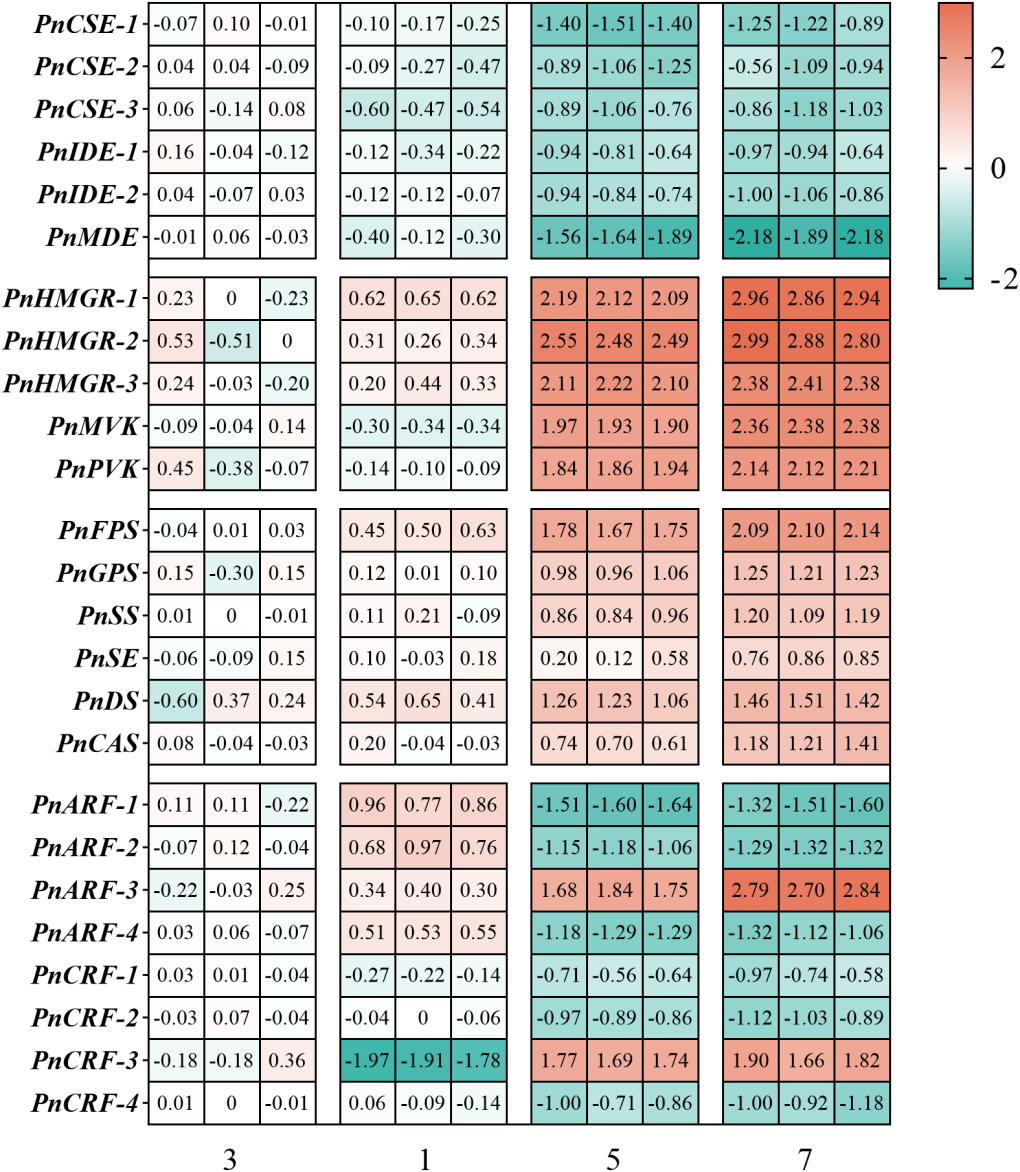
Shows the expression levels of various genes in the of *P. notoginseng* anther callus, as determined by RT-qPCR. The figure includes TCA cycle related genes *PnCSE-1* (citrate synthase 1), *PnCSE-2* (citrate synthase 2), *PnCSE-3* (citrate synthase 3), *PnIDE-1* (isocitrate dehydrogenase 1), *PnIDE-2* (isocitrate dehydrogenase 1), *PnMDE* (malate dehydrogenase), MVA pathway related genes *PnHMGR-1*, *PnHMGR-2*, *PnHMGR-3*, *PnMVK* and *PnPVK*, saponin biosynthesis pathway related genes *PnFPS*, *PnGPS*, *PnSS*, *PnSE*, *PnDS*, *PnCAS*, auxin response factor related genes *PnARF-1* (Auxin response factor 1), *PnARF-2* (Auxin response factor 2), *PnARF-3* (Auxin response factor 3)*, PnARF-4* (Auxin response factor 4), and cytokinin response factor related genes *PnCRF-1* (Cytokinin response factor 1), *PnCRF-2* (Cytokinin response factor 2), *PnCRF-3* (Cytokinin response factor 3)*, PnCRF-4* (Cytokinin response factor 4). Red indicates high gene expression, while blue indicates low gene expression.

Furthermore, RT-qPCR was performed to analyze the expression of genes related to the TCA cycle and saponin synthesis. The results showed that hormone combination No.5 and No.7 significantly increased the expression of MVA pathway-related genes (*PnHMGR-1*, *PnHMGR-2*, *PnHMGR-3*, *PnMVK*, *PnPVK*) and saponin synthesis pathway genes (*PnFPS*, *PnGPS*, *PnSS*, *PnSE*, *PnDS*, *PnCAS*) in anther callus, with combination No.7 showing the strongest induction effect. In contrast, combinations No.1 and No.3 exhibited lower expression levels of these genes. As for TCA cycle-related genes (*PnCSE-1*, *PnCSE-2*, *PnCSE-3*, *PnIDE-1*, *PnIDE-2*, *PnMDE*), hormone combination No.1 and No.3 significantly increased their expression, with combination No.3 showing the highest induction effect. However, these genes were expressed at lower levels in hormone combinations No.5 and No.7 (**Figure 9**).

### 3.8 *PnARF-3* and *PnCRF-3* regulate the distribution of Acetyl-CoA

ARFs and CRFs are key regulatory elements downstream of auxin and cytokinin signaling pathways. Since these factors vary among different hormone combinations, we measured their expression levels in *P. notoginseng* anther callus. It was found that *PnARF-1*, *PnARF-2*, *PnARF-4*, *PnCRF-1*, *PnCRF-2* and *PnCRF-4* were highly expressed in the hormone combination No.3 and No.1, with their levels positively correlating with the concentrations of added auxin and cytokinin. Additionally, *PnARF-3* and *PnCRF-3* exhibited higher response levels in hormones combination No.5 and No.7 (**Figure 9**). Similarly, the exogenous application of hormone combinations No.5 and No.7 was able to significantly induce high expression level of *PnARF-3* and *PnCRF-3* in annual *P. notoginseng* plants (**Supplementary Figure 2**). Based on these observations, we hypothesize that high levels of *PnARF-1*, *PnARF-2*, *PnARF-4*, *PnCRF-1*, *PnCRF-2*, and *PnCRF-4* promote ATP production and inhibit saponin synthesis. Conversely, high levels of *PnARF-3* and *PnCRF-3* inhibit ATP production and enhance saponin synthesis.

**Figure 10:**
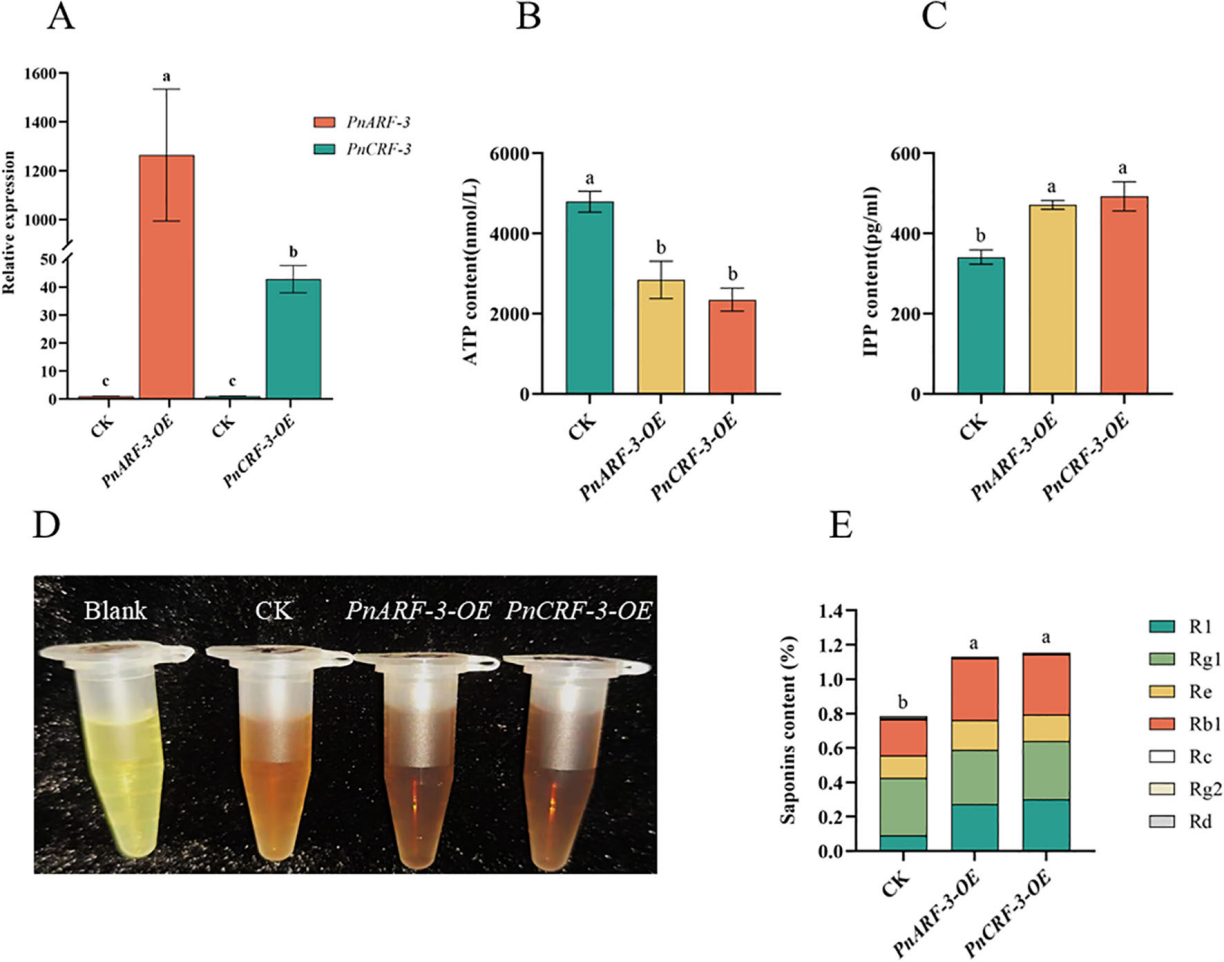
Shows various analyses of *P. notoginseng* anther callus following the overexpression of *PnARF-3* and *PnCRF-3*. **(A)** The expression levels of *PnARF-3* and *PnCRF-3* in callus of *P. notoginseng* anther after transformation were detected by RT-qPCR; **(B)** ATP content in callus of *P. notoginseng* anther after overexpression of *PnARF-3* and *PnCRF-3* was detected by kit; **(C)** IPP content in callus of *P. notoginseng* anther after overexpression of *PnARF-3* and *PnCRF-3* was detected by kit; **(D)** Total saponins content in callus of *P. notoginseng* anther after overexpression of *PnARF-3* and *PnCRF-3* was measured by kit; **(E)** The contents of total saponins of seven monomers in callus of *P. notoginseng* anther after overexpression of *PnARF-3* and *PnCRF-3* were detected by HPLC. Data are expressed as average values ± SE (n=3), different letters indicate significant differences at p < 0.05 (Student’s test).

**Figure 11:**
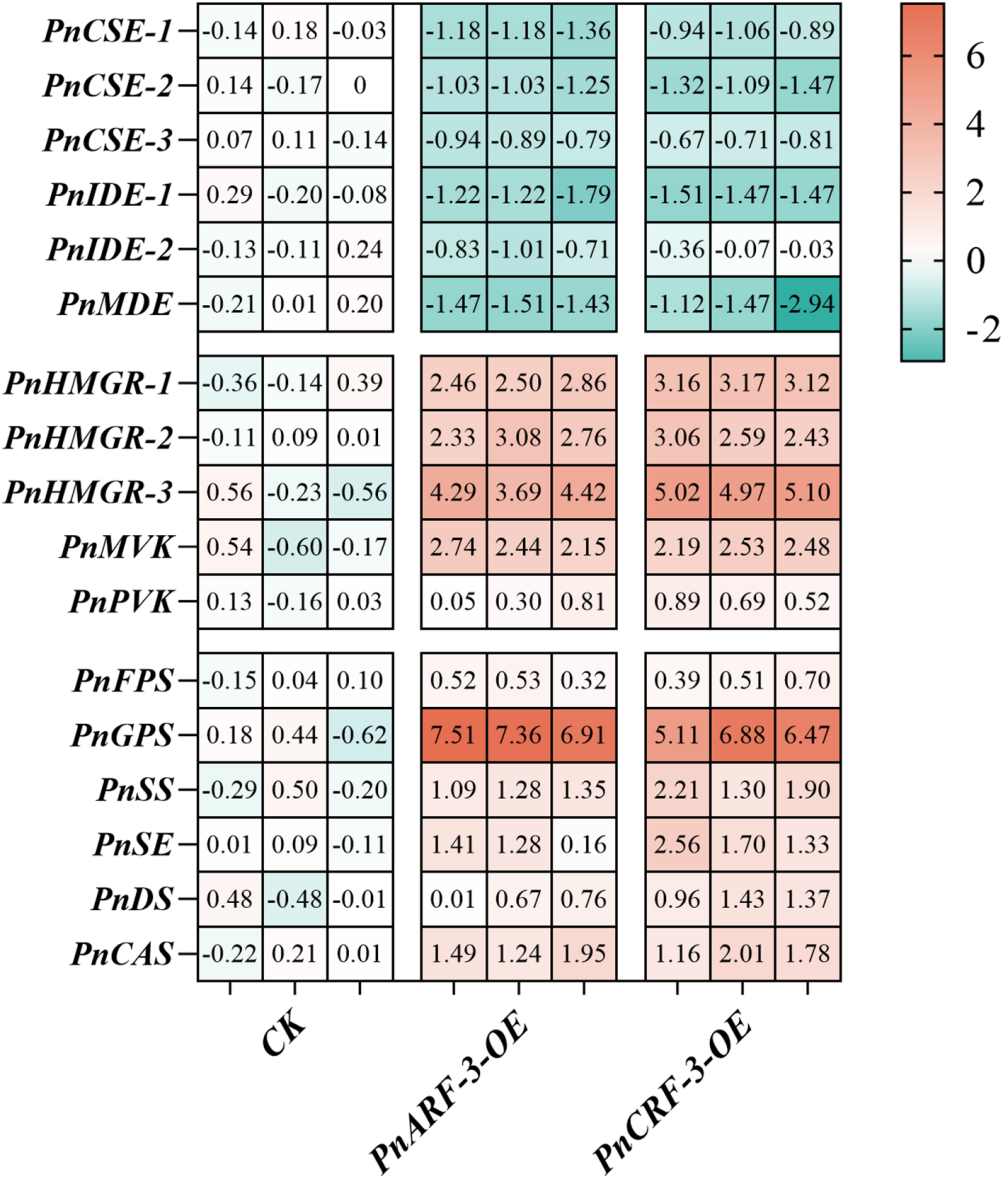
Shows the expression levels of various genes in the callus of *P. notoginseng* anther following the overexpression of *PnARF-3* and *PnCRF-3*, as determined by RT-qPCR. The genes are categorized as follows: TCA cycle related genes *PnCSE-1*, *PnCSE-2*, *PnCSE-3, PnIDE-1*, *PnIDE-2*, *PnMDE*, MVA pathway related genes *PnHMGR-1*, *PnHMGR-2*, *PnHMGR-3*, *PnMVK*, *PnPVK*, saponin biosynthesis pathway related genes *PnFPS*, *PnGPS*, *PnSS*, *PnSE*, *PnDS* and *PnCAS.* Red indicates high gene expression, while blue indicates low gene expression.

Furthermore, *PnARF-3* and *PnCRF-3* were overexpressed in *P. notoginseng* anther callus. The results showed that, compared with the control callus, the expression level of *PnARF-3* and *PnCRF-3* were significantly increased in the transformed callus (**Figure 10A**), confirming that *PnARF-3* and *PnCRF-3* were successfully expressed in *P. notoginseng* anther callus. The expression levels of MVA pathway-related genes (*PnHMGR-1*, *PnHMGR-2*, *PnHMGR-3*, *PnMVK*, *PnPVK*) and saponin synthesis-related genes (*PnGPS*, *PnFPS*, *PnCAS*, *PnSS*, *PnSE*, *PnDS*) in *P. notoginseng* anther callus overexpressing *PnARF-3* and *PnCRF-3* were higher than those in control callus. Conversely, the expression levels of TCA cycle-related genes (*PnCSE-1*, *PnCSE-2*, *PnCSE-3*, *PnIDE-1*, *PnIDE-2*, *PnMDE*) were lower in the overexpressed callus compared to the control (**Figure 11**). Meanwhile, the ATP content in callus overexpressing *PnARF-3* and *PnCRF-3* was significantly lower than in control callus (**Figure 10B**), with ATP levels was 59% and 49% lower, respectively. The callus transformed by overexpression of *PnARF-3* and *PnCRF-3* exhibited a darker coloration in the saponin colorimetric assay, indicating a higher content of total saponins (**Figure 10D**). The contents of IPP (**Figure 10C**) and total saponins of the seven monomers (**Figure 10E**) were also significantly increased compared to the control callus. Specifically, IPP levels were 38% and 44% higher, respectively, while the total saponin content of the seven monomers was 42% and 45% higher, respectively. Among these, notoginsenoside R1 and ginsenoside Rb1 were significantly increased. These results demonstrate that overexpression of *PnARF-3* and *PnCRF-3* promotes the production of IPP, enhances the expression of genes related to the MVA pathway and saponin synthesis, and consequently increase the saponin content. This overexpression also inhibits the TCA cycle, leading to reduced ATP production.

## 4 Discussion

During the process of callus induction, various studies have shown that plant callus induction is influenced by multiple factors, including explant type and exogenous hormones (Luo et al., 2018; Deng et al., 2020; Dai et al., 2020). In particular, different proportions of auxin and cytokinin not only influence the success rate of callus induction but also determine the type of callus (Isah et al., 2018; Maadon et al., 2016). In *Sinopodophyllum emodi* (Guo et al., 2023), callus induced from sterile seedlings successfully formed embryogenic callus under the hormone combination of 1.0 mg/L 6-BA, 0.1 mg/L NAA, 0.1 mg/L GA_3_. In contrast, non-embryogenic callus was primarily induced under the MS medium with 1.0 mg/L 6-BA, 0.2 mg/L NAA, 0.1 mg/L GA_3_. In *Angelica sinensis* (Huang et al., 2023), embryogenic callus was effectively induced under the combination of 2.0 mg/L IBA and 0.2 mg/L KT, while non-embryogenic callus was induced under the combination of 1.5 mg/L 2,4-D, 0.4 mg/L NAA, and 0.4 mg/L 6-BA. This finding is consistent with our results, which showed that the combinations of 2,4-D + 6-BA and 2,4-D + NAA + KT induced embryogenic callus (**Figures 3F–I**) with robust meristematic activity, providing potential for differentiation into embryoid bodies (Li et al., 2019). In contrast, the combination of 2,4-D + KT + IAA and 2,4-D + 6-BA + NAA + KT predominantly induced non-embryogenic callus (**Figures 3A–D**), which exhibited minimal division capability (Aini et al., 2017). At the same time, it was observed that hormone concentration did not affect the callus type, different hormone combinations influenced it (**Supplementary Figure 1**).

Plant hormones are an effective means to regulate plant growth and production of secondary metabolites (Wang et al., 2021). Plant hormones are classified into five categories: auxin, gibberellin (GA), cytokinin, abscisic acid (ABA) and ethylene (Santner et al., 2009). Extensive research has established that auxins primarily regulate cell elongation, while cytokinins stimulate cell division and regulate the absorption, transport, assimilation, and metabolism of nutrients. Various combinations of plant hormones can promote callus growth during plant tissue culture (Chapman and Estelle, 2009; Mok and Mok, 2001; El-Showk et al., 2013; Abualia et al., 2023). In *Azadirachta indica* (Ashokhan et al., 2020), both TDZ and 2,4-D were found to impact the growth and development of callus. Using *A. indica leaves* as explants, after 14 days of culture, 0.6 mg/L TDZ alone induced 3.38 ± 0.08 g of green callus, while 0.6 mg/L 2,4-D alone induced 0.76 ± 0.03 g of brown callus. However, the combined use of 0.6 mg/L TDZ and 0.6 mg/L 2,4-D yielded only 0.16 ± 0.02g green callus, indicating that different hormones had significant effects on callus growth. This study primarily examined the impact of auxins and cytokinins on *P. notoginseng* anther callus under various combinations. It was found that the ratio of different hormone types significantly affected the relative growth rate of *P. notoginseng* anther callus, whereas the concentration of the same type of hormone did not significantly influence the relative growth rate (**Figure 4B**).

Acetyl-CoA acts as a common initiator for both the TCA cycle and the MVA pathway. The TCA cycle primarily produces the primary metabolite ATP, while the MVA pathway predominantly generates terpenoid secondary metabolites, such as ginsenoside Rd and ginsenoside Rg1. Due to this shared initiator, ATP and certain terpene secondary metabolites exhibit an antagonistic relationship due to their competition for Acetyl-CoA. Qi et al. (2017) used sterile seedlings of *P. notoginseng* as explants to induce callus. In their study on the effects of hormone ratio, nitrogen source, and light on the accumulation of saponins in callus, they found that the hormone ratio had the greatest effect. Li et al. (2010) demonstrated that when the callus proliferation rate was the highest, the accumulation of secondary metabolite rosemary was inhibited. These studies suggest that in the process of callus induction, different hormone ratios affect the production of ATP and secondary metabolites, while ATP will affect the relative growth rate of callus. This is consistent with our findings, which showed that the combination of 2,4-D + 6-BA and 2,4-D + NAA + KT were most beneficial for the accumulation of saponin content in *P. notoginseng* anther callus. But these combinations also resulted in a slower relative growth rate, potentially inhibiting the TCA cycle. In contrast, the relative growth rate was faster with the combinations of 2,4-D + KT + IAA and 2,4-D + 6-BA + NAA + KT (**Figure 4B**), which promoted the TCA cycle but inhibited the MVA pathway, thereby reducing saponin content accumulation in the callus (**Figure 5B**). These results indicated that hormone combination promoting the growth of *P. notoginseng* callus inhibited secondary metabolites production, indicating a negative correlation between the accumulation of PNS and cell growth rate.

Auxin plays a critical role in various physiological and developmental processes, including organogenesis and cell elongation (Kasahara, 2016; Aloni et al., 2006). It was found that the addition of auxin IBA and NAA could increase the accumulation of saponins in suspended cells of *P. ginseng* (Jeong et al., 2009). In the culture system of *mulberry tree* callus and adventitious roots, the addition of IAA not only promoted callus growth but also increased rutin accumulation (Lee et al., 2011). ARFs respond to the concentration of auxin. The response of auxin depends on its concentration and the specificity of auxin signal networks in different cells and tissues. Paponov et al. (2008) found that ARFs responded to different concentrations of auxin, ranging from 0.1 μM to 10 μM. And the response of *ARF4*, *ARF16* and *ARF19* increased with auxin concentration, but only *ARF19* was sensitive to low auxin levels. In this study, *PnARF-1*, *PnARF-2* and *PnARF-4* in *P. notoginseng* anther callus were up-regulated in response to auxin concentration, while *PnARF-3* was down-regulated (**Figure 9**), suggesting that *PnARF-3* may be sensitive to lower auxin levels. Wang et al. (2023) demonstrated that the interaction between ARF and the transcription factor (*ERF108*) could mediate the biosynthesis of secondary cell walls in cotton fibers, consistent with our observations. After overexpression of *PnARF-3*, the content of saponins in callus was significantly increased (**Figure 10E**).

Cytokinin is an important plant hormone that participates in plant growth and development (Rashotte et al., 2006). Kinetin (KT) plays a significant role in the induction and cultivation of callus in *Ginkgo biloba* (Zhu et al., 2008) and *Bupleurum scorzonerifolium* (Cheng et al., 2015). The interaction between exogenous cytokinins and ethene enhances the accumulation of amorphine in *Catharanthus roseus* cells (Yahia et al., 1998). CRFs responds to the concentration of cytokinin and have multiple biological functions. Different CRFs exhibit varied responses to cytokinin concentrations. Hughes et al. (2020) found that *CRF6* exhibited primarily negative response to cytokinins and was sensitive to low concentrations of cytokinins. In this study, it was observed that the response of *PnCRF-1*, *PnCRF-2* and *PnCRF-4* to cytokinin concentration in *P. notoginseng* anther callus was positive, while the response of *PnCRF-3* to cytokinin concentration was negative (**Figure 9**).

**Figure 12:**
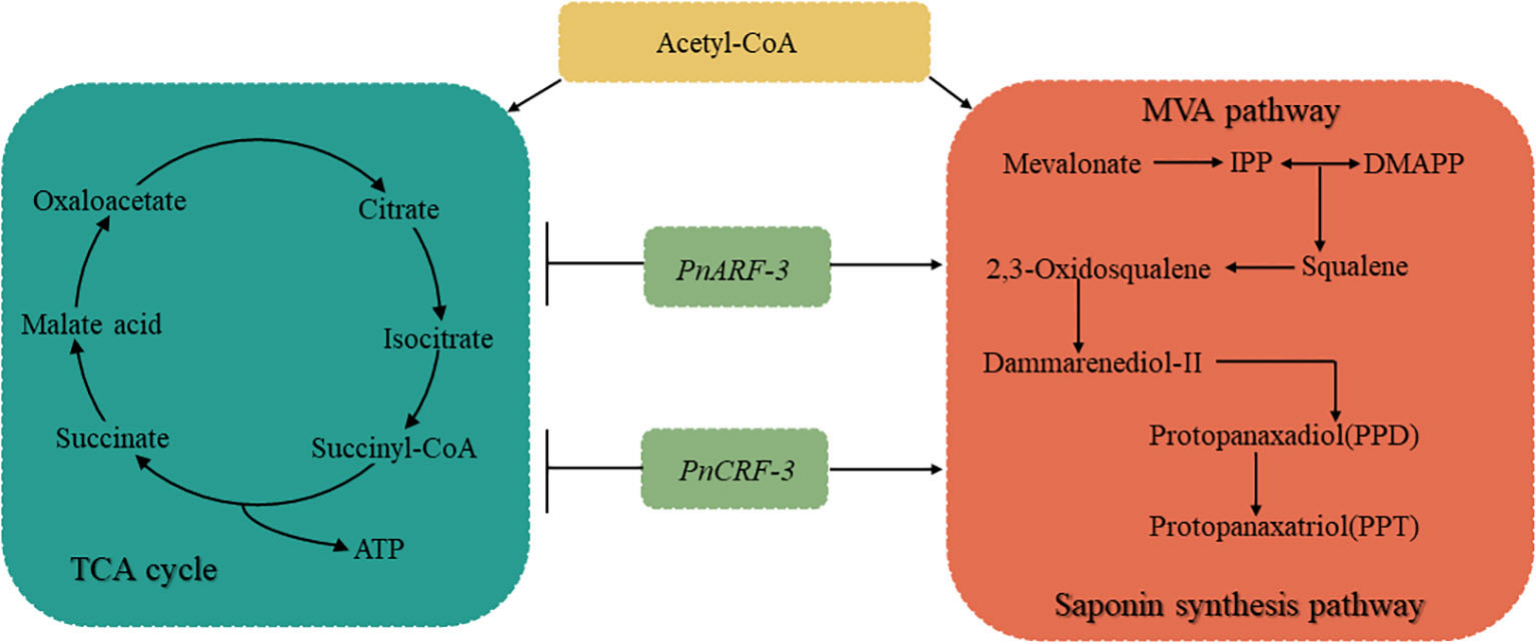
Illustrates different hormone combinations affect TCA cycle, MVA pathway and saponin synthesis pathway in *P. notoginseng* anther callus through *PnARF-3* and *PnCRF-3.* Different hormone combinations affect the expression levels of *PnARF-3* and *PnCRF-3* in the callus, and overexpression of *PnARF-3* and *PnCRF-3* leads to a reduction in the entry of Acetyl-CoA into the TCA cycle. This results in decreased ATP synthesis and a slower growth rate. Concurrently, more Acetyl-CoA is directed into the MVA pathway, which promotes the synthesis of IPP and enhances saponin accumulation. In the figure, the blue box represents the TCA cycle, while the red box highlights the MVA pathway and saponin synthesis pathway.

This study demonstrated that different hormone ratios elicited to differential responses from ARFs and CRFs. Furthermore, specific hormone combinations modulate the distribution of Acetyl-CoA through *PnARF-3* and *PnCRF-3*. *PnARF-3* and *PnCRF-3* facilitated the influx of Acetyl-CoA into the MVA pathway, thereby increasing the biosynthesis of isopentenyl pyrophosphate (IPP) and saponins. Concurrently, these transcription factor inhibit the diversion of Acetyl-CoA into the TCA cycle, resulting in decreased ATP production (**Figure 12**).

## 5 Conclusions

In the process of callus induction of *P. notoginseng* anther, different hormone combinations affected the contents of primary and secondary metabolites in callus, and the response levels to *PnARF* and *PnCRF* were also different. *PnARF-3* and *PnCRF-3* responded to low concentrations of auxin and cytokinin, affecting the distribution of Acetyl-CoA. The overexpression of *PnARF-3* and *PnCRF-3* led to increase the content of IPP and saponins, promoted the expression of genes involved in the MVA pathway such as *PnHMGR*, *PnMVK*, *PnPVK* and promoted the expression of saponin synthesis related genes including *PnFPS*, *PnGPS*, *PnSS*, *PnSE*, *PnDS*, *PnCAS*. Concurrently, ATP production decreased, and the expression of TCA cycle related genes, such as *PnCSE*, *PnIDE* and *PnMDE* was inhibited. These findings provide a scientific foundation for improving the yield and quality of *P. notoginseng* through breeding.

## Data Availability

The original contributions presented in the study are included in the article/**Supplementary Material**. Further inquiries can be directed to the corresponding authors.

## Supplementary material

Supplementary Figure 1 Results of paraffin section of anther callus of *Panax notoginseng* with different hormone combinations.

Supplementary Figure 2 Expression levels of *PnARF-3* and *PnCRF-3* in annual *P. notoginseng* under four different hormone combinations.

Supplementary Method S1 Provides detailed information on the extraction, detection, identification, quantification and statistics of differential metabolites.
