# Supplementary figures and images for: The effects of different hormone combinations on the growth of Panax notoginseng anther callus based on metabolome analysis
Source: Front Plant Sci. 2024 Dec 9;15:1503931. doi: 10.3389/fpls.2024.1503931 (PMC11667561; doi:10.3389/fpls.2024.1503931)

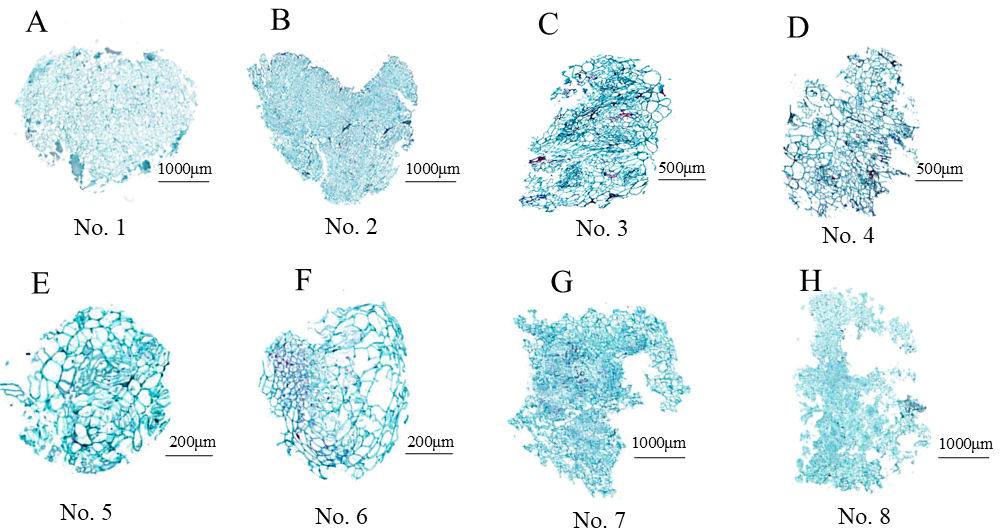

Supplement: Supplementary Figure 1 — Results of paraffin section of anther callus of Panax notoginseng with different hormone combinations. [file Image1.tif]

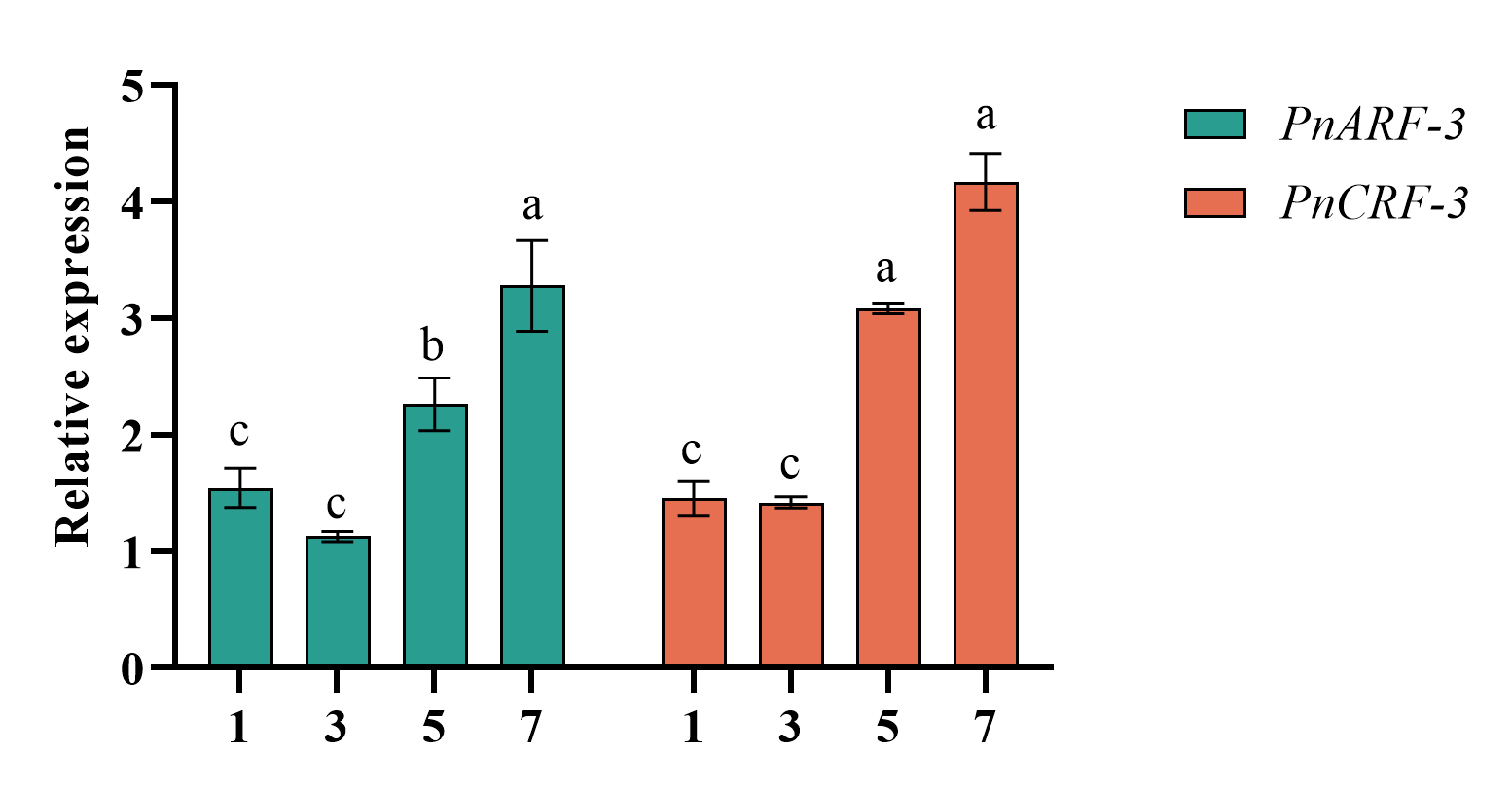

Supplement: Supplementary Figure 2 — Expression levels of PnARF-3 and PnCRF-3 in annual P. notoginseng under four different hormone combinations. [file Image2.tif]
