# Supplementary material for: The effects of different hormone combinations on the growth of Panax notoginseng anther callus based on metabolome analysis
Source: Front Plant Sci. 2024 Dec 9;15:1503931. doi: 10.3389/fpls.2024.1503931 (PMC11667561; doi:10.3389/fpls.2024.1503931)
Supplement: Supplementary Method S1 — Provides detailed information on the extraction, detection, identification, quantification and statistics of differential metabolites. [file DataSheet1.docx]

**Supplemental Method S1** **Metabolome detection**

1、Sample preparation and extraction

The metabolites were extracted and analyzed by Beijing Novogene Technology limited company. Weigh 100 mg of *Panax notoginseng* anther callus samples in different hormone combinations respectively. Place them in EP tubes and add 300ul ul of an 80% methanol aqueous solution. Vortex shake the mixture and let it rest in an ice bath for 3 minutes. Centrifuge at 13,000 r, 4 ℃ for 20 minutes. Take a certain amount of supernatant and dilute it with mass spectrometry-grade water to obtain a concentration of 32% methanol. Centrifuge again at 13,000 r, 4 ℃ for another 20 minutes to collect the supernatant which will be analyzed by LC-MS.

2、Metabolome testing condition

Chromatographic conditions：XselectHSST2（2.3 μm，2.1×130 mm）,The mobile phase was 0.1 % formic acid water (A)-0.1 % formic acid acetonitrile,gradient elution: 0-13 min，2%-100% B；13 min-17.1 min，100%~2% B；17.1 min-20 min，2% B. The column temperature was 30℃, the sample size was 4ul, and the flow rate was 0.4 mL/min.

Mass spectrometry conditions: positive ion mode: CurtainGas: 23psi, CollisionGas: Medium, IonSprayVoltage: 3300V, Temperature: 330℃, IonSourceGas1:50, IonSourceGas2：50. Negative ion mode: CurtainGas: 23 psi, CollisionGas: Medium, IonSprayVoltage: -4300V, Temperature: 330℃, IonSourceGas1:50, IonSourceGas2:5.

3、Identification and analysis

Based on the primary and secondary mass spectrometry data obtained by chromatography-tandem mass spectrometry, combined with the metabolite information from novogenDB (novogenedatabase) and the public database HumanMetabolomeDatabase, we realized the qualitative analysis, and detected the experimental samples based on the triple-quadruple-rod (MRM) mode. Q2 (daughter ion) to quantify the compounds, and Q1 (parent ion), Q2 (daughter ion), RT (retention time), DP (de-clustering voltage), and CE (collision energy) for qualitative analysis. SCIEXOSV1.4 software was used to open the downstream mass spectrometry file for the integration and correction work of the chromatographic peaks, and the peaks were screened according to the set information of the minimum peak height of 300, the Signal-to-noise ratio of 3, the smoothing points of 1 and other information. The peak Area (Area) of each chromatographic peak represents the relative content of the corresponding substance. Finally, the integral data of all chromatographic peak areas are derived to obtain the qualitative and quantitative results of metabolites

4、Statistical analysis of differential metabolites

For the analysis of the two groups, the differential metabolites were screened by VIP(VIP≥1) and FOLDCHANGE>1.2 and T-test (FDR)<0.05. The data are logarithmic transformed (log2) and mean centered before OPLS-DA. Identification of the metabolites of using compound and annotation data repository (http://www.kegg.jp/kegg/compound/), and to map annotation of metabolites to KEGGPathway database (<http://www.kegg.jp/kegg/pathway.html>). KEGG database was used to study the function and metabolic pathway of metabolites, and the figures in this study were obtained by GrapNDHAadprism. The cluster heat map was plotted using the online site hiplot and the metabolite data was normalized using z-score

**Supplemental Figure S1**

Supplemental Figure S1 Results of paraffin section of anther callus of *Panax notoginseng* with different hormone combinations. (A-H) The structure of *Panax notoginseng* anther callus in hormone combination No.1- No.8 was observed by microscope after paraffin section.

**Supplemental Figure S2**

Supplemental Figure S2 Expression levels of *PnARF-3* and *PnCRF-3* in annual *P. notoginseng* under four different hormone combinations.
