# Supplementary material for: The effects of different hormone combinations on the growth of Panax notoginseng anther callus based on metabolome analysis
Source: Front Plant Sci. 2024 Dec 9;15:1503931. doi: 10.3389/fpls.2024.1503931 (PMC11667561; doi:10.3389/fpls.2024.1503931)
Supplement: Supplementary file 4 [file Table1.docx]

Supplemental Table S1 Induction of anthers or pollens by different hormone combinations in medicinal plants

| Number | Medium | Hormone concentration mg/L | | | | | Sucrose | Agar | PH | Type | Plant | Induction rate | References |
| --- | --- | --- | --- | --- | --- | --- | --- | --- | --- | --- | --- | --- | --- |
|  |  | 2,4-D | 6-BA | NAA | KT | IAA |  |  |  |  |  |  |  |
| 1 | MS | 1.5 | — | — | 1.0 | 1.0 | 3.0% | 0.8% | 5.8-6.0 | solidity | *Panax ginseng* anther | 33.80% | [11] |
| 2 | MS | 1.5 | — | — | 0.5 | 1.0 | 3.0% | 0.8% | 5.6-6.0 | solidity |  | 24.8% |  |
| 3 | MS | 0.75 | 0.75 | 0.4 | 1.0 | — | 3.0% | 0.7% | 5.8 | solidity | *Alfalfa* pollen | 83.33% | [13] |
| 4 | MS | 1.0 | 0.75 | 0.4 | 1.0 | — | 3.0% | 0.7% | 5.8-6.0 | solidity |  | 88.33% |  |
| 5 | MS | 0.5 | 0.5 | — | — | — | 3.0% | 0.7% | 5.8-6.0 | solidity | *Macleaya cordata* anther | 26.80% | [14] |
| 6 | MS | 2.0 | 1.0 | — | — | — | 3.0% | 0.7% | 5.8 | solidity | *Angelica dahurica* anther | 38.89% | [15] |
| 7 | MS | 1.0 | — | 0.2 | 0.25 | — | 3.0% | 0.7% | 5.8 | solidity | *Forsythia suspensa* pollen | 33.33% | [16] |
| 8 | MS | 0.5 | — | 0.1 | 0.25 | — | 3.0% | 0.7% | 5.8 | solidity |  | 30.00% |  |
| 9 | MS | 2.0 | 0.5 | — | — | 3.0 | 3.0% | — | 5.6-5.8 | liquid | *Panax quinquefolius* anther | 45.63% | [12] |
| 10 | MS | 3.0 | 1.0 | — | — | 1.0 | 3.0% | — | 5.8 | liquid |  | 55.60% |  |
| 11 | MS | — | 1.0 | 0.1 | — | — | 3.0% | 0.7% | 5.8-6.0 | solidity | *Lycium barbarum* anther | 20.70% | [17] |
| 12 | 1/3MS | — | 0.5 | 2.0 | — | — | 3.0% | 0.7% | 5.8 | solidity | *Weigela florida 'Red Prince'* anther | 57.45% | [18] |
