# Supplementary material for: The effects of different hormone combinations on the growth of Panax notoginseng anther callus based on metabolome analysis
Source: Front Plant Sci. 2024 Dec 9;15:1503931. doi: 10.3389/fpls.2024.1503931 (PMC11667561; doi:10.3389/fpls.2024.1503931)
Supplement: Supplementary file 5 [file Table2.docx]

Supplemental Table S2 Primer sequence

| Gene | Forward Primer (5’- 3’) | Reverse Primer (5’- 3’) |
| --- | --- | --- |
| *PnACTIN2* | TCCAAGGGTGAATATGATGAATCG | AACCTCTCCAAAGAGAATTTCTGAGT |
| *PnGPS* | GCCGTTTGGGTTTGAACTAC | CTCGCCTTCCTTCTTCTCAC |
| *PnFPS* | ACTCAACGACCCTGCTTTC | TCAATAACAGACAGCCCTCG |
| *PnCAS* | ATCCAGGCAGTGTAGTCTTAC | CAGGCATTAGAAACATAGGACC |
| *PnSS* | CTATGTAGCAGGACTTGTTGG | ATGCGTGACTTTGGTATCTC |
| *PnSE* | TTTGATTACCTGAGCCTCG | CCAACGCCATAAATAGCC |
| *PnDS* | AGATAGAGGATTCTGTGGCG | CGAACTGCTTCACTGTTGTC |
| *PnHMGR-1* | TAAGAGAAATTGGAGGGCAGGATGG | CAGGAGGTCTTCTAGCAGCATGAG |
| *PnHMGR-2* | CGATGACGACGAGGAGGATGTATG | CTGTGATTGAGCCACTGGAGGAG |
| *PnHMGR-3* | CACTCCTCTACTCTCAAAGCCTCTG | CACTCCTCTACTCTCAAAGCCTCTG |
| *PnMVK* | TCGCTGCCTCCATTGATCTATACAC | GCATCCTTGATCCTTTGAACTGTCC |
| *PnPVK* | AGGTGTCAGATGCGGCAGATG | CCAGGAACTCCAGCCAACAGG |
| *PnCSE-1* | ACATTGGCGAGAATCCCTGGATG | CCGACACCTGACCTAGCTTATCTG |
| *PnCSE-2* | CCTCGGATGGCTGGCTACTTATC | CTCACGCTGTGGCATGTAGGG |
| *PnCSE-3* | TCCCCTCCAGCAATCTGTCTTATTC | AATGTCTAGCACACGAGCCAGTC |
| *PnIDE-1* | TGACTTCAGTCCTGGTTTGTCCTG | ACGCTATGCTGTTTGTGCTAGTTTC |
| *PnIDE-2* | CTAGTTGTCAAAGGACCTGGAAAGC | AGCAAAAGCACGAATGGACTCATC |
| *PnMDE* | TGTTGCCACGACGAATGTTGTTG | CCTTCCTCTCCATTCCATCCTTCC |
| *PnARF-1* | AAAGAGACAGGGAAACGGCTACAG | CTCCACTGCTGCTCCAGACAC |
| *PnARF-2* | GACTGCTTGAGGATTTGGTGGTTG | AGTTGGGTTGTTCGTGTTTGATTCG |
| *PnARF-3* | AGCCAAGGACGAGTCAGTTCATC | GCCCAACTATTGTGCCCATAAACC |
| *PnARF-4* | GATGCTGGGGAATTGCGTGTTG | ATGCTCTGGCTGGATATGACTGATG |
| *PnCRF-1* | TCCATTAAGACGGGTACGGGTTTG | TCAGGACCACGCAACTGTATAGC |
| *PnCRF-2* | AAGAAGCGGAAGAGTGTCAGTCAG | ACGGATGAAACGGTTCGGACTC |
| *PnCRF-3* | ATCAAGCGACGAAGAGGAGACAC | GCCAGCCGCTATTAAGGTTTGC |
| *PnCRF-4* | TGCTACGGTTCAGTGCTCAGTC | TGTCCCTGGTCGGTTCATTAACTG |
