# Supplementary material for: The effects of different hormone combinations on the growth of Panax notoginseng anther callus based on metabolome analysis
Source: Front Plant Sci. 2024 Dec 9;15:1503931. doi: 10.3389/fpls.2024.1503931 (PMC11667561; doi:10.3389/fpls.2024.1503931)
Supplement: Supplementary file 6 [file Table3.docx]

Supplemental Table S3 Induction of callus in anther of *Panax notoginseng*

with different hormone combinations

| Hormone combination | 1 | 2 | 3 | 4 | 5 | 6 | 7 | 8 | 9 | 10 | 11 | 12 |
| --- | --- | --- | --- | --- | --- | --- | --- | --- | --- | --- | --- | --- |
| Induced condition | √ | √ | √ | √ | √ | √ | √ | √ | × | × | × | × |
