# Supplementary material for: The effects of different hormone combinations on the growth of Panax notoginseng anther callus based on metabolome analysis
Source: Front Plant Sci. 2024 Dec 9;15:1503931. doi: 10.3389/fpls.2024.1503931 (PMC11667561; doi:10.3389/fpls.2024.1503931)
Supplement: Supplementary file 7 [file Table4.docx]

Supplemental Table S4 Specific contents of 7 monomer saponins in anther callus of

*Panax notoginseng* with different hormone combinations

| Different hormone combinations | Notoginsenoside R1 (%) | Ginsenoside Rg1 (%) | Ginsenoside Re (%) | Ginsenoside Rb1 (%) | Ginsenoside Rc (%) | Ginsenoside Rg2 (%) | Ginsenoside Rd (%) | Total (%) |
| --- | --- | --- | --- | --- | --- | --- | --- | --- |
| 1 | 0.0624 | 0.3001 | 0.1987 | 0.2607 | 0.0038 | 0.0002 | 0.0231 | 0.8491 |
| 2 | 0.5945 | 0.3720 | 0.4197 | 0.2750 | 0.0031 | 0 | 0.0007 | 1.6650 |
| 3 | 0.0899 | 0.3338 | 0.1430 | 0.2117 | 0.0076 | 0.0090 | 0.0004 | 0.7953 |
| 4 | 0.2240 | 0.5027 | 0.4271 | 0.4028 | 0.0072 | 0.0001 | 0.0008 | 1.5648 |
| 5 | 0.3538 | 0.4331 | 0.5696 | 0.8269 | 0.0106 | 0.0428 | 0.0002 | 2.2369 |
| 6 | 0.4172 | 0.4456 | 0.5395 | 0.3711 | 0.0037 | 0.0311 | 0.0005 | 1.80886 |
| 7 | 0.5917 | 0.6815 | 0.8834 | 0.6905 | 0.00634 | 0.0069 | 0.0020 | 2.8624 |
| 8 | 0.3946 | 0.4509 | 0.7358 | 0.4344 | 0.0041 | 0.0041 | 0.0006 | 2.0245 |
